# Metal-dependent base pairing of bifacial iminodiacetic acid-modified uracil bases for switching DNA hybridization partner†

**DOI:** 10.1039/d2sc06534g

**Published:** 2023-01-03

**Authors:** Keita Mori, Yusuke Takezawa, Mitsuhiko Shionoya

**Affiliations:** a Department of Chemistry, Graduate School of Science, The University of Tokyo 7-3-1 Hongo, Bunkyo-ku Tokyo 113-0033 Japan takezawa@chem.s.u-tokyo.ac.jp shionoya@chem.s.u-tokyo.ac.jp

## Abstract

Dynamic control of DNA assembly by external stimuli has received increasing attention in recent years. Dynamic ligand exchange in metal complexes can be a central element in the structural and functional transformation of DNA assemblies. In this study, *N*,*N*-dicarboxymethyl-5-aminouracil (dcaU) nucleoside with an iminodiacetic acid (IDA) ligand at the 5-position of the uracil base has been developed as a bifacial nucleoside that can form both hydrogen-bonded and metal-mediated base pairs. Metal complexation study of dcaU nucleosides revealed their ability to form a 2:1 complex with a Gd^III^ ion at the monomeric level. The characteristics of base pairing of dcaU nucleosides were then examined inside DNA duplexes. The results revealed that the formation of the metal-mediated dcaU–Gd^III^–dcaU pair significantly stabilized the DNA duplex containing one dcaU–dcaU mismatch (Δ*T*_m_ = +16.1 °C). In contrast, a duplex containing a hydrogen-bonded dcaU–A pair was destabilized in the presence of Gd^III^ (Δ*T*_m_ = −3.5 °C). The Gd^III^-dependent base pairing of dcaU bases was applied to control the hybridization preference of DNA in response to metal ions. The hybridization partner of a dcaU-containing strand was reversibly exchanged by the addition and removal of Gd^III^ ions. Since the incorporation of a single dcaU base can switch the hybridization behavior of DNA, the bifacial dcaU base would be a versatile building block for imparting metal responsiveness to DNA assemblies, allowing the rational design of dynamic DNA systems.

## Abstract 2

A novel *N*,*N*-dicarboxymethyl-5-aminouracil (dcaU) nucleobase was found to form both a hydrogen-bonded dcaU–A and a metal-mediated dcaU–Gd^III^–dcaU base pair. The hybridization partner of the dcaU-containing DNA was altered in response to Gd^III^ ions.

## Graphical abstract

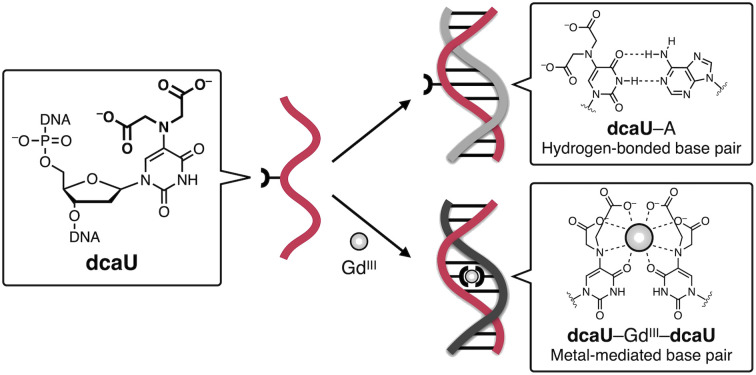

## Introduction

Sequence-dependent self-assembly of DNA molecules has been extensively applied to the construction of precisely defined nanoarchitectures.^1,2^ In addition, dynamic control of DNA assembly enables highly programmable mechanical motions and functional switching, which have been demonstrated in DNA-based nanodevices, molecular machines, and more advanced systems.^3–5^ In such systems, oligonucleotides with a specific sequence are utilized as input signals to dynamically control DNA hybridization basically through toehold-mediated DNA strand displacement reactions.^6,7^ Efforts are also being focused on developing DNA molecular systems that respond to external stimuli^8^ such as light irradiation^9–11^ and pH changes.^12–14^ Among a variety of stimuli, metal complexation can be a versatile trigger for structural and functional transformation of DNA assemblies because the reaction can be reversible and selective if the right combination of ligands and metal ions is chosen.^15–17^ Metal-mediated artificial base pairing, in which two ligand-bearing nucleosides and a specific metal ion form a 2:1 complex, has been regarded as a useful structural motif that confers metal responsiveness to DNA molecules.^18–21^ Metal-mediated base pairing inside DNA duplexes typically causes great thermal stabilization of the duplex due to the formation of stable coordination bonds in addition to hydrogen bonds between canonical nucleobases. The metal-mediated base pairing has been applied not only to the construction of metallo-DNA hybrid structures,^22–27^ but also to the development of metal-responsive DNA molecular machines^28,29^ and catalytically active DNAs (DNAzymes).^30–34^

Aiming for efficient switching of DNA structures in response to metal ions, we focused on noncanonical 5-modified uracil (**U**^**X**^) nucleobases with a metal-binding functional group (X) at the 5-position. The **U**^**X**^ bases are expected to exhibit a “bifacial” base-paring behavior, forming hydrogen-bonded **U**^**X**^–A base pairs and metal-mediated **U**^**X**^–M–**U**^**X**^ base pairs (M = metal ion) in the absence and presence of certain metal ions, respectively. Our previous studies on 5-hydroxyuracil (**U**^**OH**^)^35–37^ and 5-carboxyuracil (**caU**)^38^ have shown that DNA duplexes with **U**^**X**^–**U**^**X**^ mismatches are thermally stabilized by forming metal-mediated base pairs such as **U**^**OH**^–Gd^III^–**U**^**OH**^ and **caU**–Cu^II^–**caU**, while duplexes containing **U**^**X**^–A pairs are destabilized by the addition of metal ions. Thus, **U**^**X**^ bases would be excellent building blocks for constructing metal-responsive DNA systems. However, the metal-dependent stabilization of duplexes requires the tandem introduction of multiple **U**^**OH**^–**U**^**OH**^ or **caU**–**caU** mismatch pairs in the DNA sequence.^35,38^ Therefore, the flexibility of sequence design was greatly limited when these 5-modified uracil bases were applied to metal-responsive DNA systems.

**Fig. 1:**
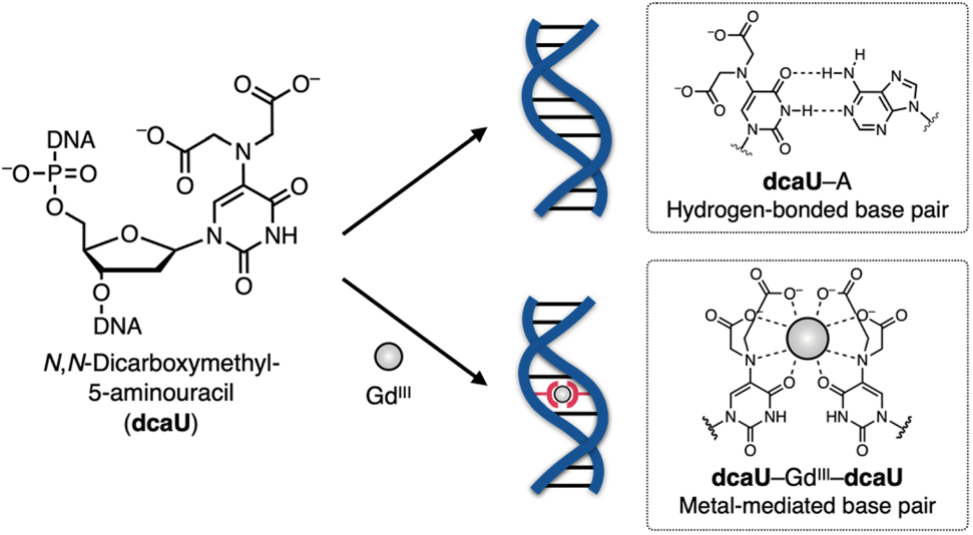
Schematic representation of Gd^III^-dependent base-pairing behaviors of bifacial *N*,*N*-dicarboxymethyl-5-aminouracil (dcaU) nucleobases.

In this study, we have developed a new bifacial nucleobase, an *N*,*N*-dicarboxymethyl-5-aminouracil (dcaU) nucleobase that stably forms a metal-mediated single base pair within DNA duplexes (Fig. 1). Here, a tridentate iminodiacetic acid (IDA) ligand was introduced at the 5-position of the uracil base. The IDA ligand is known to form stable complexes with various transition metals and lanthanide ions^39–45^ and is a substructure of chelating ligands such as EDTA (ethylenediaminetetraacetic acid) and BAPTA (1,2-bis(2-aminophenoxy)ethane-*N*,*N*,*N*′,*N*′-tetraacetic acid).^46^ The IDA and the 4-carbonyl group of the dcaU base were expected to provide a tetradentate chelating environment to form metal-mediated base pairs. A hydrogen-bonded dcaU–A base pair can also be formed because the hydrogen-bonding site remains unchanged. Thus, metal-mediated switching between the two base pairing modes was expected. To create stable metal-mediated base pairs, we employed Gd^III^, a lanthanide ion suitable for forming a multicoordinated dcaU–Gd^III^–dcaU complex.^47,48^ Due to the chelating effect and negative charges of the dcaU bases, it was expected to complex with Gd^III^ more efficiently than the previously reported **U**^**OH**^–Gd^III^–**U**^**OH**^ base pairing. We investigated the metal-dependent base-pairing behaviors of bifacial dcaU bases within DNA duplexes and further applied them to metal-responsive switching of the DNA hybridization partner.

## Results and discussion

### Synthesis of dcaU nucleosides and dcaU-containing DNA strands

**Scheme 1:**
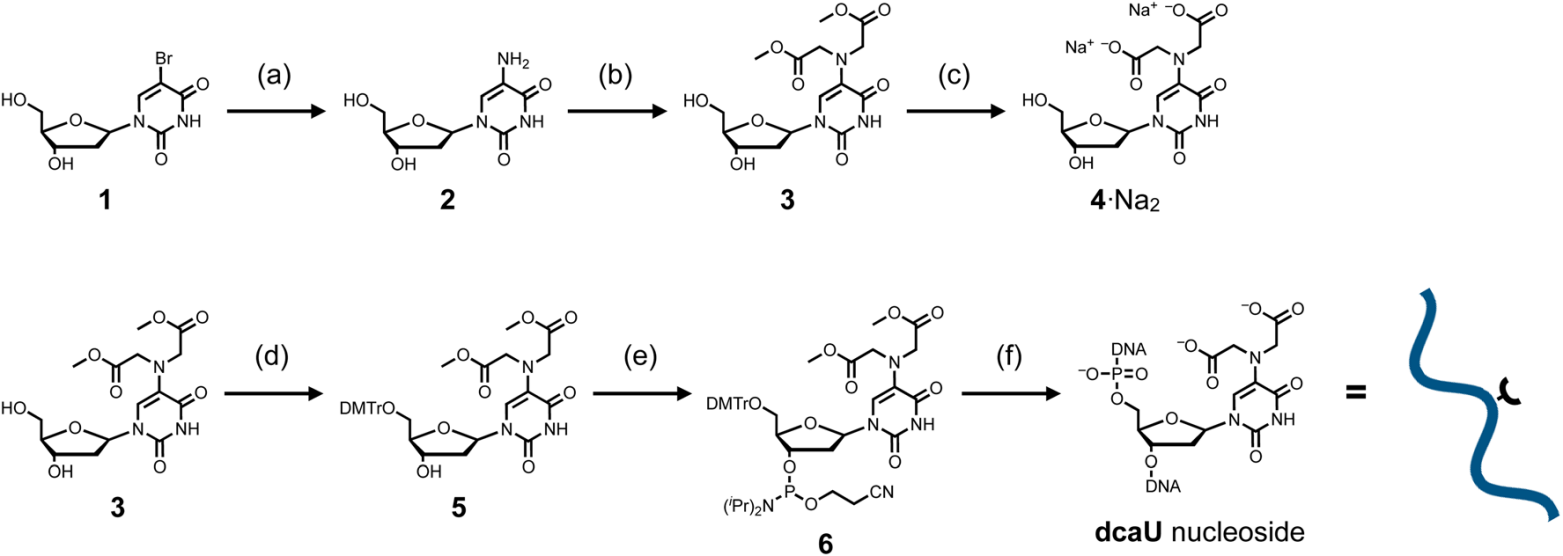
Synthetic route for dcaU nucleoside and dcaU-containing DNA strands. (a) (i) Benzylamine, 90 °C; (ii) H_2_, Pd/C, MeOH, 25 °C. (b) Methyl bromoacetate, 2,6-lutidine, MeOH, 60 °C, 38% in 2 steps. (c) 0.1 M NaOH aq, 60 °C, quant. (d) DMTrCl, DMAP, pyridine, rt, 59%. (e) NC(CH_2_)_2_OP[N(^*i*^Pr)_2_]_2_, diisopropylamine, 1*H*-tetrazole, CH_2_Cl_2_, rt, 65%. (f) Solid-phase DNA synthesis by an automated synthesizer. Synthesized DNA strands were cleaved from the solid support and deprotected by 0.3 M NaOH aq at 37 °C. DMTr = 4,4′-dimethoxytrityl, ^*i*^Pr = isopropyl.

The dcaU nucleoside was synthesized from commercially available 5-bromo-2′-deoxyuridine (1) (Scheme 1). The 5-bromo group was replaced by an amino group to afford 5-amino-2′-deoxyuridine (2).^49^ To introduce the IDA ligand moiety, crude nucleoside 2 was then reacted with methyl bromoacetate at 60 °C in the presence of 2,6-lutidine. As a result, the 5-amino group was selectively functionalized, yielding a crude mixture containing the desired disubstituted nucleoside 3 and the monosubstituted byproduct. After purification by column chromatography, methyl-protected dcaU nucleoside 3 was obtained in two steps in 38% yield. The methyl ester group was deprotected by treatment with 2 equiv. of NaOH at 60 °C to give dcaU nucleoside 4 as the disodium salt (4·Na^+^_2_), which was used for metal complexation studies (see below).

The dcaU nucleoside was then incorporated into DNA strands by standard solid-phase DNA synthesis. The methyl-protected nucleoside 3 was reacted with DMTrCl to protect the 5′-hydroxy group to give nucleoside 5 in 59% yield. Subsequent reaction with 2-cyanoethyl-*N*,*N*,*N*′,*N*′-tetraisopropylphosphorodiamidite in the presence of diisopropylammonium tetrazolide gave the phosphoramidite derivative 6 in 65% yield, which was immediately used for DNA synthesis using an automated synthesizer. Two complementary 15-mer DNA strands containing one dcaU nucleotide in the middle were synthesized to investigate metal complexation of dcaU bases inside a duplex (Table 1). DNA synthesis was performed with an extended coupling time for dcaU nucleosides. All the DNA strands were cleaved from the solid support and deprotected with 0.3 M NaOH aqueous solution. dcaU-containing oligonucleotides were purified by reverse-phase HPLC (Fig. S1, ESI†) and characterized by ESI-TOF mass spectrometry.

**Table 1:** Sequences of DNA strands used in this study

DNA	Sequences^a^ (5′ to 3′)
**1**	CAC ATT AdcaUT GTT GTA
**1T**	CAC ATT ATT GTT GTA
**2**	TAC AAC AdcaUT AAT GTG
**2N** (**N** = A, T, G, C)	TAC AAC A**N**T AAT GTG
**2A′**	TAC AAC AAT AAT G

a dcaU: *N*,*N*-dicarboxymethyl-5-aminouracil.

### Metal complexation of dcaU nucleosides

**Fig. 2:**
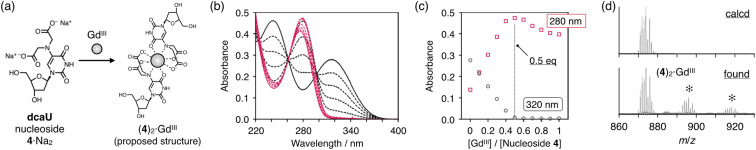
Metal complexation of dcaU nucleoside. (a) Scheme. (b) UV-vis absorption spectra of nucleoside 4 in the presence of different concentrations of Gd^III^ ions. [Nucleoside 4] = 200 μM, [Gd^III^]/[4] = 0 (black solid line), 0.1, 0.2, 0.3, 0.4 (black dotted lines), 0.5 (red solid line), 0.6, 0.7, 0.8, 0.9, and 1.0 (red dotted lines) in 10 mM HEPES buffer (pH 7.0), *l* = 0.3 cm, 25 °C. (c) Plot of the absorbance at 280 nm and 320 nm against the ratio of [Gd^III^]/[nucleoside 4]. (d) ESI mass spectrum of nucleoside 4 with 0.5 equiv. of Gd^III^ ions. Small signals (*) were attributed to sodium adducts. [Nucleoside 4] = 100 μM, [Gd^III^]/[4] = 0.50 in 10 mM NH_4_OAc buffer (pH 7.0), positive mode.

The dcaU nucleoside was expected to form stable metal complexes because of its IDA-like chelating ligand moiety. Coordination of the IDA moiety as well as the 4-carbonyl group was expected to form an octacoordinated 2:1 complex with lanthanide ions. The metal complexation property of dcaU was first examined with monomeric nucleoside 4 and Gd^III^ ions (Fig. 2a). We analyzed changes in UV-vis absorption spectra while varying amount of Gd^III^ ions (Fig. 2b). In the absence of Gd^III^, two absorption bands were observed near 240 nm and 320 nm. When Gd^III^ ions were added, the intensity of these bands decreased and a new absorption band appeared near 280 nm. The absorption spectra varied linearly in the range [Gd^III^]/[dcaU nucleoside] = 0 to 0.5, with isosbestic points at 261 and 297 nm. The absorbance at 280 nm was maximal in the presence of 0.5 equiv. of Gd^III^ ions (Fig. 2c). These results indicate that a 2:1 complex of dcaU nucleosides and a Gd^III^ ion is quantitatively formed. Job's plot analysis further confirmed the 2:1 metal complexation (Fig. S2†). Moreover, ESI-TOF mass spectrometry provided the expected 2:1 complex signal (found: 874.07 (*z* = +1); calcd for [(4)_2_ + Gd^III^ + 2H]^+^: 874.10), confirming the stable formation of dcaU–Gd^III^–dcaU base pair at the monomeric level (Fig. 2d and S3†).

The UV absorption spectra of the dcaU nucleoside showed changes similar to the BAPTA ligand upon metal complexation.^46^ BAPTA has been reported to form octacoordinated metal complexes involving the coordination of three oxygen atoms and an aniline-type N atom. Therefore, it was inferred that the dcaU nucleobase functions as a tetradentate ligand coordinating to a Gd^III^ ion, forming an octacoordinated 2:1 complex. This coordination structure is in good agreement with the properties of lanthanide ions, which generally accommodate high coordination numbers. The 2:1 complexation of the dcaU nucleosides and a Gd^III^ ion may indicate metal-mediated base pairing within the DNA duplexes.

### Gd^III^-mediated base pairing of dcaU nucleobases within DNA duplexes

**Fig. 3:**
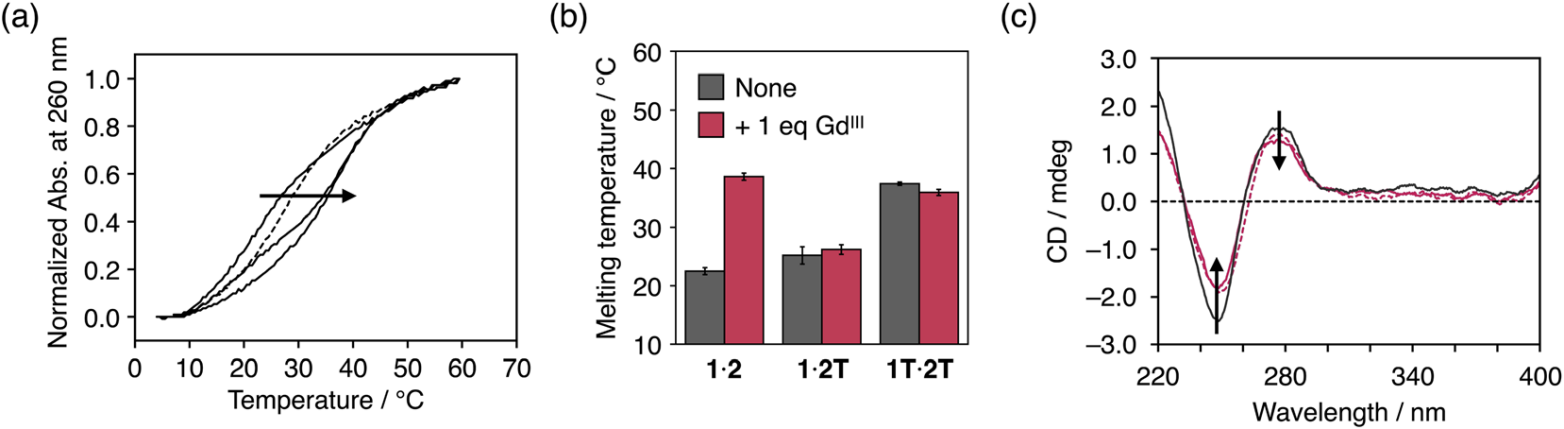
(a) Melting curves of duplex **1**·**2** containing a dcaU–dcaU mismatch in the presence of different concentrations of Gd^III^ ions. [Duplex] = 2.0 μM, [Gd^III^]/[duplex] = 0, 0.5, 1 (solid lines), and 2 (dotted line) in 10 mM HEPES buffer (pH 7.0), 100 mM NaCl, 0.2 °C min^−1^. (b) Melting temperatures of duplexes containing a dcaU–dcaU, a dcaU–T, and a T–T mispair in the absence and presence of Gd^III^ ions. (c) CD spectra of duplexes containing a T–A (black solid line), a dcaU–dcaU (red solid line), and a dcaU–Gd^III^–dcaU pair (red dotted line). [Duplex] = 2.0 μM, 10 mM HEPES buffer (pH 7.0), 100 mM NaCl, *l* = 0.3 cm, 5 °C.

Metal complexation of dcaU bases within DNA duplexes was studied using DNA duplex **1**·**2** containing one dcaU–dcaU mismatch in the middle. Melting analysis of duplex **1**·**2** was performed in the absence and presence of Gd^III^ ions (Fig. 3a). Under conditions without Gd^III^ ions, the melting temperature (*T*_m_) of duplex **1**·**2** was 22.5 °C, which is significantly lower than that of the natural full-match duplex **1T**·**2A** containing a T–A pair instead of the dcaU–dcaU (*T*_m_ = 47.9 °C). This result indicates that the dcaU–dcaU behaves as a mismatch inside the duplex. The *T*_m_ of duplex **1**·**2** is lower than that of duplex **1T**·**2T** containing a T–T mismatch pair (37.4 °C). This is thought to be due to the electrostatic repulsion between the dcaU bases with negatively charged carboxylates, similar to the 5-carboxyuracil^38^ and 4-carboxyimidazole^32,50–52^ nucleobases reported previously.

The addition of 1 equiv. of Gd^III^ ions markedly improved the thermal stability of duplex **1**·**2** (*T*_m_ = 38.6 °C, Δ*T*_m_ = +16.1 °C) (Fig. 3a). On the other hand, neither duplex 1·2T with a dcaU–T mismatch nor duplex **1T**·**2T** with a T–T mispair showed any changes in the duplex stability with the addition of Gd^III^ (Fig. 3b and S4†). These results suggest that the thermal stabilization of duplex **1**·**2** resulted from the formation of an interstrand complex between the two dcaU bases and a Gd^III^ ion, namely, the dcaU–Gd^III^–dcaU base pair. Titration of Gd^III^ ions confirmed the stoichiometry of the complexation between dcaU and Gd^III^. Further melting experiments were performed with various amounts of Gd^III^ ions (Fig. 3a). When 0.5 equiv. of Gd^III^ ions was added, the melting curve showed a two-step transition, suggesting the formation of both Gd^III^-free and Gd^III^-containing duplexes. The addition of 2 equiv. of Gd^III^ ions shifted the melting curve to the lower temperature side compared to the melting curve in the presence of an equimolar amount of Gd^III^ ions. This result indicates that the addition of excess amounts of Gd^III^ ions destabilized duplex **1**·**2**. This may be because the Gd^III^ ions form a 1:1 complex with each dcaU base and are unable to crosslink the duplex. These results suggest a 2:1 stoichiometry of the metal complexation and support the quantitative formation of a single dcaU–Gd^III^–dcaU base pair inside the DNA duplex.

Fig. 3c shows the circular dichroism (CD) spectra of duplex **1**·**2** in the absence and presence of 1 equiv. of Gd^III^ ions. The CD spectra exhibit a positive Cotton effect at 279 nm and a negative Cotton effect at 249 nm, which are characteristic of B-type right-handed DNA duplexes. Compared to duplex **1T**·**2A** with a canonical T–A pair, duplex **1**·**2** showed weaker CD intensity. This is likely due to structural distortion caused by the introduction of the dcaU–dcaU mismatch pair. The CD spectra of duplex **1**·**2** were not significantly changed by the addition of Gd^III^ ions. This result suggests that the dcaU–Gd^III^–dcaU base pair was accommodated inside the DNA duplex without significant distortion, even though the Gd^III^ complex adopts a relatively bulky nonplanar structure.^53^

It is also worth mentioning that other transition metal ions such as Fe^III^, Ni^II^, Cu^II^, and Zn^II^ did not show stabilization of duplex **1**·**2** (Fig. S5†), indicating good metal selectivity of dcaU. Since dcaU functions as a tetradentate ligand, lanthanide ions, which can adopt a high coordination number, are suitable for the formation of metal-mediated dcaU–M–dcaU base pairs through 2:1 metal complexation. The 3+ valent Gd^III^ ion may also be important for compensating the negative charges of the dcaU–dcaU mispair.

Importantly, the degree of stabilization of duplex **1**·**2** by Gd^III^ (Δ*T*_m_ = +16.1 °C) was similar to that observed in our previous work with a duplex containing three **U**^**OH**^–Gd^III^–**U**^**OH**^ pairs (Δ*T*_m_ = +18.3 °C).^35^ This may be due to the tetradentate coordination ability of the dcaU bases. The large duplex stabilization caused by single dcaU–Gd^III^–dcaU base pairing may be useful for metal-dependent control of DNA assembly.

### Gd^III^-induced thermal destabilization of duplexes containing a dcaU–A pair

**Fig. 4:**
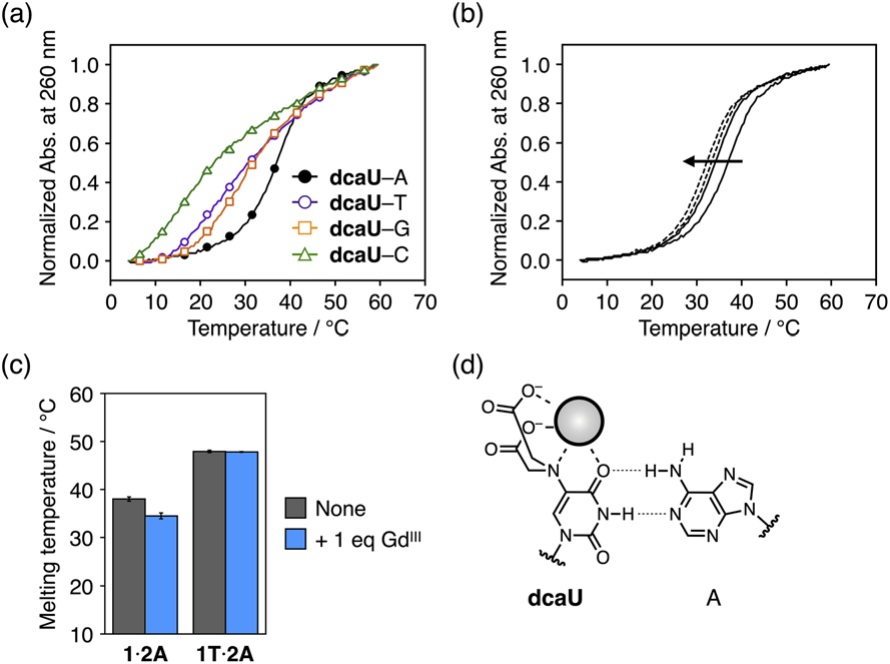
(a) Melting curves of duplexes containing a dcaU–N pair (N = A, T, G, or C). [Duplex] = 2.0 μM in 10 mM HEPES buffer (pH 7.0), 100 mM NaCl, 0.2 °C min^−1^. (b) Melting curves of duplex **1·2A** containing a dcaU–A pair in the presence of different concentrations of Gd^III^ ions. [Duplex] = 2.0 μM, [Gd^III^]/[duplex] = 0, 1 (solid lines), 2, and 4 (dotted lines) in 10 mM HEPES buffer (pH 7.0), 100 mM NaCl, 0.2 °C min^−1^. (c) Melting temperatures of duplexes containing a dcaU–A (**1·2A**) and a T–A pair (**1T**·**2A**) in the absence and presence of Gd^III^ ions. (d) A proposed structure of the dcaU–A pair with weakened hydrogen bonding in the presence of Gd^III^ ions.

The dcaU base was expected to form a base pair with a natural adenine (A) base within DNA duplexes because it retains the hydrogen-bonding site of the canonical thymine or uracil base. Therefore, to investigate the base pairing properties of dcaU with natural nucleobases, we performed melting experiments of duplexes containing a dcaU–N pair (N = A, T, G, or C) (Fig. 4a). The duplex **1**·**2A** with a dcaU–A pair was found to have higher thermal stability (*T*_m_ = 38.0 °C) than the others. This result suggests that the dcaU base forms a hydrogen-bonded base pair with the natural A base in a manner similar to the Watson–Crick T–A base pair. It should be noted that the thermal stability of duplex **1**·**2A** is lower than that of the natural duplex **1T**·**2A** containing a T–A pair (47.9 °C). This may be attributed to the electrostatic repulsion between the negatively charged dcaU base and phosphate backbones.

The addition of 1 equiv. of Gd^III^ ions decreased the thermal stability of duplex **1**·**2A** (*T*_m_ = 34.5 °C, Δ*T*_m_ = −3.5 °C) (Fig. 4b). The addition of more than 1 equiv. of Gd^III^ further destabilized the duplex. In contrast, the addition of Gd^III^ ions did not affect the stability of the natural duplex **1T**·**2A** (Fig. 4c and S6†). These results indicate that the binding of Gd^III^ ions to the dcaU base weakened the hydrogen bonding of the dcaU–A pair (Fig. 4d).

Thus, the thermal stability of DNA duplexes containing dcaU bases was found to be Gd^III^-dependent. Under Gd^III^-free conditions, duplex **1**·**2A** with a dcaU–A pair (*T*_m_ = 38.0 °C) was found to be much more stable than duplex **1**·**2** with a dcaU–dcaU mismatch (22.5 °C). In the presence of equal amounts of Gd^III^ ions, the dcaU–Gd^III^–dcaU pair formation and destabilization of the dcaU–A pair resulted in higher stability of duplex **1**·**2** (38.6 °C) than duplex **1**·**2A** (34.5 °C). The metal-mediated changes in stability of the two duplexes (**1**·**2** and **1**·**2A**) would be applicable to the dynamic switching of DNA structures in response to metal ions.

### Gd^III^-dependent switching of hybridization partner of dcaU-containing DNA strands

**Fig. 5:**
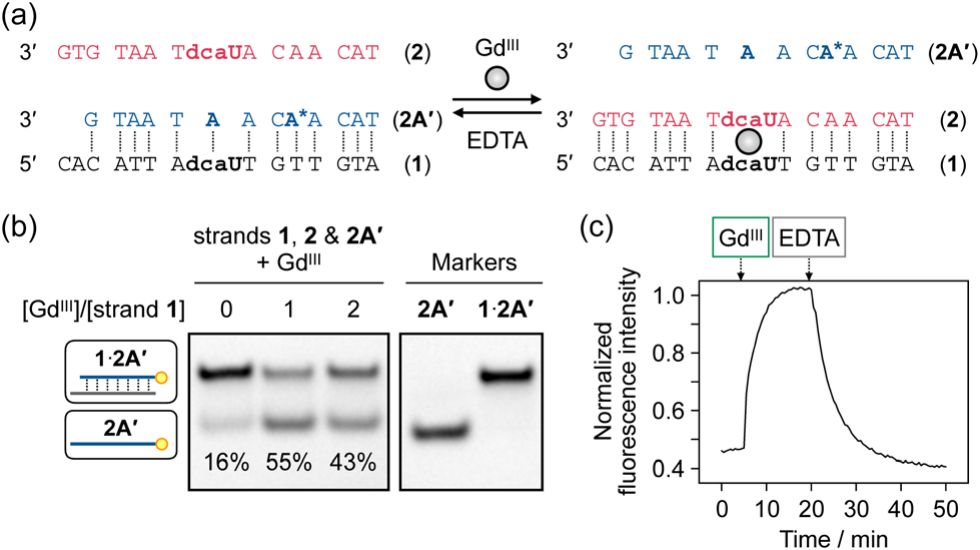
Gd^III^-dependent switching of the hybridization partner of dcaU-containing strand 1. (a) Sequence design. (b) Native PAGE analysis of an equimolar mixture of strands 1, 2, and 2A′ in the absence and presence of Gd^III^ ions (1 equiv.). The samples were annealed prior to the analysis. Strand 2A′ was labeled with FAM at its 5′ end for detection. 20% gel at 4 °C. The yields were calculated from the average of three independent experiments. (c) Time-course fluorescence analysis of Gd^III^-dependent hybridization behaviors of dcaU-containing strand 1. An adenine base (A*) of strand 2A′ was replaced by 2-aminopurine. After annealing the mixture of the three strands, Gd^III^ (1 equiv.) and EDTA (5 equiv.) were added sequentially. *λ*_ex_ = 303 nm, *λ*_em_ = 371 nm, 20 °C. [DNA] = 0.5 μM each in 10 mM HEPES buffer (pH 7.0), 100 mM NaCl.

The dcaU base was thus found to form both a metal-mediated dcaU–Gd^III^–dcaU base pair and a hydrogen-bonded dcaU–A base pair within the DNA duplexes. We then applied this bifacial base-pairing behavior of dcaU bases to switching the DNA hybridization partner in response to Gd^III^ ions. Hybridization preference of dcaU-containing strand 1 was studied in the absence and presence of Gd^III^ ions (Fig. 5a). DNA strand 1 was mixed with two complementary strands, 2 and 2A′, containing one central dcaU and A base, respectively. To facilitate the strand exchange, the length of strand 2A′ was designed to be two-base shorter than the others. Duplex **1·2A′** was expected to be formed by dcaU–A base pairing and duplex **1**·**2** by metal-mediated dcaU–Gd^III^–dcaU pairing in the presence of Gd^III^ ions. After annealing the mixture, the hybridization products were analyzed by native polyacrylamide gel electrophoresis (PAGE), for which strand 2A′ was labeled with a fluorophore (FAM) (Fig. 5b). The result showed that in the absence of Gd^III^ ions, duplex **1·2A′** containing a dcaU–A pair was predominantly formed. Upon addition of 1 equiv. of Gd^III^ ions, the release of strand 2A′ was observed, indicating instead the formation of duplex **1**·**2** with a Gd^III^-mediated dcaU–Gd^III^–dcaU base pair. Furthermore, the formation of duplex **1**·**2** was confirmed by staining the gel with SYBR Gold (Fig. S7†). These results indicate that the hybridization partner of strand 1 was switched by the addition of Gd^III^ ions. In addition, the addition of an excess of Gd^III^ ions decreased the yield of duplex **1**·**2**. These results confirm the stoichiometric response of the oligonucleotides containing dcaU and are in good agreement with the Gd^III^-dependent thermal stabilization of duplex **1**·**2** discussed above.

Furthermore, it was demonstrated that the hybridization preference can be controlled in a Gd^III^-responsive manner under isothermal conditions. To evaluate the hybridization behavior, 2-aminopurine (AP) was introduced into strand 2A′. It is well known that the fluorescence emission from the AP base is quenched when it forms a base pair with a T base in a DNA duplex.^54,55^ Thus, the release of strand 2A′ can be detected by an increase in AP fluorescence (Fig. 5c). Addition of 1 equiv. of Gd^III^ ions to the pre-annealed mixture of strands 1, 2, and 2A′ resulted in an immediate increase in fluorescence intensity, indicating that duplex **1·2A′** dissociated to release strand 2A′ and yield duplex **1**·**2** with a dcaU–Gd^III^–dcaU pair. A chelating agent EDTA was then added to dissociate the Gd^III^ ions from the DNA. Addition of excess EDTA (5 equiv.) resulted in a gradual decrease in luminescence intensity, confirming the formation of the original duplex **1·2A′**. These results indicate that the hybridization preference of the dcaU-containing strand is reversibly switched. Furthermore, sequential addition and removal of Gd^III^ ions resulted in repeated exchange of the hybridization partner of strand 1, albeit with gradually decreasing efficiency (Fig. S8†). Thus, the bifacial base-pairing property of the dcaU base was successfully used to reversibly control DNA hybridization in response to metal ions, even under isothermal conditions.

## Conclusions

In this study, we have developed an *N*,*N*-dicarboxymethyl-5-aminouracil (dcaU) nucleobase with an iminodiacetic acid (IDA) ligand at the 5 position as a bifacial uracil analog that can form both metal-mediated and hydrogen-bonded base pairs. The dcaU nucleoside was synthesized from 5-bromodeoxyuridine in good yield. UV–vis titration experiments and mass spectrometry revealed that the dcaU nucleosides form a 2:1 complex with a Gd^III^ ion. The DNA duplex containing a dcaU–dcaU mismatch was found to be significantly stabilized (Δ*T*_m_ = +16.1 °C) by the addition of equimolar Gd^III^ ions, due to the formation of a metal-mediated dcaU–Gd^III^–dcaU base pair. In contrast, the duplex containing a dcaU–A pair was thermally destabilized by the addition of Gd^III^ ions, possibly due to the weakening of the hydrogen bond between dcaU and A as a result of the binding of Gd^III^ to the dcaU base. This metal-mediated nature of bifacial base-pairing was further exploited to control the hybridization preference of dcaU-containing DNA strands in a metal-responsive manner. It was demonstrated that the addition and removal of Gd^III^ ions reversibly exchanged the hybridization partner of dcaU-containing strand under isothermal conditions. Therefore, this unique base pairing behavior of dcaU is expected to have further applications in the dynamic control of DNA supramolecules stimulated by metal ions.

Most metal-mediated base pairs have a planar structure that is compatible with a base pair stacking environment. Although the dcaU–Gd^III^–dcaU base pair probably takes on a nonplanar and bulky structure, it can be incorporated into the DNA duplexes without significant structural distortion and induces duplex stabilization. Our previous studies on bidentate 5-hydroxyuracil (**U**^**OH**^) bases^35^ required consecutive incorporation of **U**^**OH**^–Gd^III^–**U**^**OH**^ pairs for thermal stabilization of DNA duplexes. In contrast, the present study demonstrates that only one dcaU–Gd^III^–dcaU pair is capable of significant stabilization of the duplex. These results indicate that the introduction of the multidentate IDA ligand improves the coordination ability of the dcaU base to Gd^III^ compared to the **U**^**OH**^ base. As a result, the dcaU–Gd^III^–dcaU base pair will have sufficient stability to compensate for destabilization due to structural distortion. This unprecedented feature of the dcaU–Gd^III^–dcaU base pairing broadly expands the chemical diversity of metal-mediated artificial base pairing. Since the 5-position of uracil and cytosine bases can be modified with a variety of metal-coordinating functional groups, the metal selectivity and coordination affinity of the 5-modified pyrimidines may be tunable. Thus, the 5-modification of pyrimidine bases may be a powerful way to develop metal-responsive nucleobase analogs.

Metal-mediated control of the structure and function of DNA assemblies is generally based on the stabilization of metal-containing states.^56–59^ In contrast, metal-dependent hybridization demonstrated in this study stands on the switching between two different states involving hydrogen-bonded base pairs (dcaU–A) and metal-mediated base pairs (dcaU–Gd^III^–dcaU). We believe that metal-dependent switching of the base pairing properties of the 5-modified nucleobases will be a versatile tool for efficient switching of DNA nanostructures. Since only single incorporation of dcaU base allows for metal-dependent exchange of the hybridization partner, it is possible to construct metal-responsive DNA assemblies by simply replacing any T base with dcaU. Thus, metal-dependent base pairing of bifacial dcaU bases makes it possible to rationally design dynamic DNA systems, such as molecular machines, that function in response to specific metal ion stimuli.

## Data availability

All the data supporting this study are included in the main text and the ESI.†

## Author contributions

Y. T. and M. S. conceived and directed the study. K. M. performed the experiments and analyzed the data with the aid of Y. T. All the authors prepared the manuscript.

## Conflicts of interest

There are no conflicts to declare.

## Supplementary Material

SC-014-D2SC06534G-s001

## References

[cit1] Seeman N. C., Sleiman H. F. (2017). Nat. Rev. Mater..

[cit2] Hong F., Zhang F., Liu Y., Yan H. (2017). Chem. Rev..

[cit3] Ramezani H., Dietz H. (2020). Nat. Rev. Genet..

[cit4] Wang F., Liu X., Willner I. (2015). Angew. Chem., Int. Ed..

[cit5] Murata S., Toyota T., Nomura S. M., Nakakuki T., Kuzuya A. (2022). Adv. Funct. Mater..

[cit6] Zhang D. Y., Seelig G. (2011). Nat. Chem..

[cit7] Simmel F. C., Yurke B., Singh H. R. (2019). Chem. Rev..

[cit8] Lu S., Shen J., Fan C., Li Q., Yang X. (2021). Adv. Sci..

[cit9] Wang C., O'Hagan M. P., Li Z., Zhang J., Ma X., Tian H., Willner I. (2022). Chem. Soc. Rev..

[cit10] Lubbe A. S., Szymanski W., Feringa B. L. (2017). Chem. Soc. Rev..

[cit11] Kamiya Y., Asanuma H. (2014). Acc. Chem. Res..

[cit12] Hu Y., Cecconello A., Idili A., Ricci F., Willner I. (2017). Angew. Chem., Int. Ed..

[cit13] Mattath M. N., Ghosh D., Pratihar S., Shi S., Govindaraju T. (2022). ACS Omega.

[cit14] He S., Ge Z., Zuo X., Fan C., Mao X. (2021). NPG Asia Mater..

[cit15] McConnell A. J., Wood C. S., Neelakandan P. P., Nitschke J. R. (2015). Chem. Rev..

[cit16] Chakrabarty R., Mukherjee P. S., Stang P. J. (2011). Chem. Rev..

[cit17] Ihara T., Kitamura Y., Katsuda Y. (2022). Life.

[cit18] Takezawa Y., Shionoya M. (2012). Acc. Chem. Res..

[cit19] Takezawa Y., Müller J., Shionoya M. (2017). Chem. Lett..

[cit20] Naskar S., Guha R., Müller J. (2020). Angew. Chem., Int. Ed..

[cit21] Müller J. (2019). Coord. Chem. Rev..

[cit22] Tanaka K., Tengeiji A., Kato T., Toyama N., Shionoya M. (2003). Science.

[cit23] Tanaka K., Clever G. H., Takezawa Y., Yamada Y., Kaul C., Shionoya M., Carell T. (2006). Nat. Nanotechnol..

[cit24] Takezawa Y., Maeda W., Tanaka K., Shionoya M. (2009). Angew. Chem., Int. Ed..

[cit25] Clever G. H., Carell T. (2007). Angew. Chem., Int. Ed..

[cit26] Johannsen S., Megger N., Böhme D., Sigel R. K. O., Müller J. (2010). Nat. Chem..

[cit27] Kondo J., Tada Y., Dairaku T., Hattori Y., Saneyoshi H., Ono A., Tanaka Y. (2017). Nat. Chem..

[cit28] Wang Z.-G., Elbaz J., Willner I. (2011). Nano Lett..

[cit29] Lu C.-H., Cecconello A., Elbaz J., Credi A., Willner I. (2013). Nano Lett..

[cit30] Takezawa Y., Nakama T., Shionoya M. (2019). J. Am. Chem. Soc..

[cit31] Nakama T., Takezawa Y., Sasaki D., Shionoya M. (2020). J. Am. Chem. Soc..

[cit32] Takezawa Y., Hu L., Nakama T., Shionoya M. (2020). Angew. Chem., Int. Ed..

[cit33] Nakama T., Takezawa Y., Shionoya M. (2021). Chem. Commun..

[cit34] Rajasree S. C., Takezawa Y., Shionoya M. Chem. Commun..

[cit35] Takezawa Y., Nishiyama K., Mashima T., Katahira M., Shionoya M. (2015). Chem.–Eur. J..

[cit36] Nishiyama K., Takezawa Y., Shionoya M. (2016). Inorg. Chim. Acta.

[cit37] Nishiyama K., Mori K., Takezawa Y., Shionoya M. (2021). Chem. Commun..

[cit38] Takezawa Y., Suzuki A., Nakaya M., Nishiyama K., Shionoya M. (2020). J. Am. Chem. Soc..

[cit39] Schmitt W., Jordan P. A., Henderson R. K., Moore G. R., Anson C. E., Powell A. K. (2002). Coord. Chem. Rev..

[cit40] Walters M. A., Vapnyar V., Bolour A., Incarvito C., Rheingold A. L. (2003). Polyhedron.

[cit41] Corradi A. B., Palmieri C. G., Nardelli M., Pellinghelli M. A., Tani M. E. V. (1973). J. Chem. Soc., Dalton Trans..

[cit42] Tribet M., Covelo B., Choquesillo-Lazarte D., González-Pérez J. M., Castiñeiras A., Niclós-Gutiérrez J. (2003). Inorg. Chem. Commun..

[cit43] Ren Y.-P., Long L.-S., Mao B.-W., Yuan Y.-Z., Huang R.-B., Zheng L.-S. (2003). Angew. Chem., Int. Ed..

[cit44] Zhang Q.-Z., He X., Yu Y.-Q., Chen S.-M., Lu C.-Z. (2005). Z. Anorg. Allg. Chem..

[cit45] Thompson L. C. (1962). Inorg. Chem..

[cit46] Tsien R. Y. (1980). Biochemistry.

[cit47] Bünzli J.-C. G. (2014). J. Coord. Chem..

[cit48] Martinez-Gomez N. C., Vu H. N., Skovran E. (2016). Inorg. Chem..

[cit49] Gourdain S., Petermann C., Harakat D., Clivio P. (2010). Nucleosides, Nucleotides Nucleic Acids.

[cit50] Sandmann N., Defayay D., Hepp A., Müller J. (2019). J. Inorg. Biochem..

[cit51] Hu L., Takezawa Y., Shionoya M. (2022). Chem. Sci..

[cit52] Hu L., Takezawa Y., Shionoya M. Chem. Commun..

[cit53] The CD spectrum of duplex **1·2** changed upon addition of 2 equiv. of Gd^III^ ions (Fig. S9†). This result indicates that the destabilization of the duplex by 2 equiv. of Gd^III^ ions is accompanied by further conformational distortion of the duplex

[cit54] Law S. M., Eritja R., Goodman M. F., Breslauer K. J. (1996). Biochemistry.

[cit55] Jean J. M., Hall K. B. (2001). Proc. Natl. Acad. Sci. U. S. A..

[cit56] Takezawa Y., Yoneda S., Duprey J.-L. H. A., Nakama T., Shionoya M. (2016). Chem. Sci..

[cit57] Takezawa Y., Sakakibara S., Shionoya M. (2021). Chem.–Eur. J..

[cit58] Ihara T., Ohura H., Shirahama C., Furuzono T., Shimada H., Matsuura H., Kitamura Y. (2015). Nat. Commun..

[cit59] Wang L.-L., Zhang Q.-L., Wang Y., Liu Y., Lin J., Xie F., Xu L. (2021). Chem. Sci..

