# Supplementary material for: Metal-dependent base pairing of bifacial iminodiacetic acid-modified uracil bases for switching DNA hybridization partner
Source: Chem Sci. 2023 Jan 3;14(5):1082–8. doi: 10.1039/d2sc06534g (PMC9891364; doi:10.1039/d2sc06534g)

Electronic Supplementary Material (ESI) for Chemical Science.
This journal is © The Royal Society of Chemistry 2023

Supplementary Information

Metal-dependent base pairing of bifacial iminodiacetic acid-modified uracil bases for switching DNA hybridization partner

Keita Mori,[a] Yusuke Takezawa*[a] and Mitsuhiko Shionoya*[a]

[a] *Department of Chemistry, Graduate School of Science, The University of Tokyo, 7-3-1 Hongo, Bunkyo-ku, Tokyo 113-0033, Japan. E-mail: takezawa@chem.s.u-tokyo.ac.jp; shionoya@chem.s.u-tokyo.ac.jp*

Contents

1. Experimental Procedures ····**S2**

2. Supplementary Figures ····**S6**

3. NMR Spectra ····**S9**

1. Experimental Procedures

General. All organic syntheses were performed under argon atmosphere with commercial dehydrated solvents (Wako Pure Chemical Industries). The reagents were purchased from Wako Pure Chemical Industries, Tokyo Chemical Industry (TCI), and Sigma Aldrich, and were used without further purification. Silica gel column chromatography was performed using Merck Silica Gel 60 (80–230 or 230–400 mesh) or amine-modified silica gel NH-DM1020 (Fuji Silysia). All NMR spectra were measured on a Bruker AVANCE 500 spectrometer (500 MHz for ^{1}H, 126 MHz for ^{13}C, and 202 MHz for ^{31}P). The spectra were referenced to tetramethylsilane (TMS) in $CDCl_3$ (δ 0 ppm) or to the residual solvent signals in CD_3OD (δ 3.31 ppm for ^{1}H, 49.00 ppm for ^{13}C). Electrospray ionization-time-of-flight (ESI-TOF) mass spectra were recorded on a Waters LCT Premier XE or a Bruker Compact System. Metal sources were purchased from FUJIFILM Wako Pure Chemical Industries ($FeCl_3\cdot6H_2O$ (99.9% purity), $NiCl_2\cdot6H_2O$ (99.9%) and $CuCl_2\cdot2H_2O$ (99.9%)) and Soekawa Chemical Co. ($ZnSO_4\cdot7H_2O$ (99.9%) and $GdCl_3\cdot6H_2O$ (99.9%)).

Compound 3. 5-Bromo-2′-deoxyuridine (768 mg, 2.50 mmol) was dissolved in benzylamine (5.5 mL, 50.0 mmol, 20 eq). After stirred at 90 °C for 3 h, the reaction mixture was concentrated under reduced pressure followed by co-evaporation with toluene. The residue was dissolved in water (100 mL) and washed with CH_2Cl_2 (100 mL). The aqueous layer was mixed with 28% NH_3 aq (25 mL) and washed again with CH_2Cl_2 (50 mL × 4). After concentrated under reduced pressure, the residue (859 mg) was dissolved in methanol (38 mL) and 10% Pd/C (266 mg, 0.25 mmol) was added. The suspension was vigorously stirred under H_2 atmosphere at room temperature for 2 h. After removing the Pd/C powder by filtration, the filtrate was concentrated under reduced pressure to afford 5-amino-2′-deoxyuridine (672 mg, containing impurities). A part of the obtained crude 5-amino-2′-deoxyuridine (120 mg, containing impurities) was dissolved in methanol (1.9 mL). After methyl bromoacetate (93.6 µL, 0.989 mmol, 2.0 eq) and 2,6-lutidine (115 µL, 0.989 mmol, 2.0 eq) were added, the reaction mixture was stirred at 60 °C for 2 h. Methyl bromoacetate (328 µL, 3.46 mmol, 7.0 eq in total) was added in four portions for over 25 h and the mixture was further stirred at 60 °C for 17 h. The solution was then concentrated under reduced pressure. The residue was purified by silica gel column chromatography ($CHCl_3$:CH_3OH = 50:1–1:1) to afford the target compound **3** as a colorless solid (65.2 mg, 0.168 mmol, 38% in two steps).

^{1}H NMR (500 MHz, CD_3OD, 300 K): δ 7.59 (s, 1H), 6.31 (dd, J = 6.7, 6.7 Hz, 1H), 4.41–4.38 (m, 1H), 4.06–3.98 (m, 4H), 3.93–3.91 (m, 1H), 3.79–3.71 (m, 2H), 3.70 (s, 6H), 2.28–2.16 (m, 2H).

^{13}C NMR (126 MHz, CD_3OD, 300 K): δ 172.82, 162.90, 151.15, 128.43, 126.09, 89.01, 86.43, 72.41, 62.97, 54.23, 52.28, 41.26.

HRMS (ESI-TOF) *m/z*: $[M + Na]^+$ calcd for $C_{15}H_{21}N_3NaO_9$ 410.1170, found 410.1189.

Compound 4·Na$^{+}_{2}$. Nucleoside **3** (13 mg, 0.034 mmol) was dissolved in 0.1 M NaOH aqueous solution (674 µL, 0.067 mmol, 2.0 eq). After incubated at 60 °C for 22 h, the solution was concentrated under reduced pressure. The disodium salt of the target compound **4** was obtained as a colorless solid (14 mg, 0.035 mmol,

quant.).

^{1}H NMR (500 MHz, CD_3OD, 300 K): δ 7.75 (s, 1H), 6.28 (dd, J = 6.5, 6.5 Hz, 1H), 4.42–4.39 (m, 1H), 3.90–3.82 (m, 2H), 3.75–3.72 (m, 1H), 3.50–3.42 (m, 4H), 2.25–2.23 (m, 2H).

^{13}C NMR (126 MHz, CD_3OD, 300 K): 177.91, 165.26, 151.47, 130.45, 127.62, 88.93, 86.54, 71.99, 62.67, 58.99, 41.38.

HRMS (ESI-TOF) *m/z*: $[M - H + 2Na]^+$ calcd for $C_{13}H_{16}N_3Na_2O_9$ 404.0676, found 404.0678.

Compound 5. Nucleoside **3** (305 mg, 0.676 mmol) and 4-dimethylaminopyridine (8.3 mg, 0.068 mmol, 0.10 eq) were dissolved in pyridine (3.4 mL). A solution of 4,4′-dimethoxytrityl chloride (412 mg, 1.22 mmol, 1.8 eq) in pyridine (3.4 mL) was added, and the mixture was stirred at room temperature for 2 h. After the addition of methanol (1.6 mL), the reaction mixture was concentrated under reduced pressure. The residue was purified by silica gel column chromatography ($CHCl_3$:CH_3OH = 100:1–10:1) to afford the target compound **5** as a colorless foam (274 mg, 0.398 mmol, 59%).

^{1}H NMR (500 MHz, CD_3OD, 300 K): δ 7.47–7.45 (m, 2H), 7.36–7.33 (m, 4H), 7.29–7.26 (m, 2H), 7.21–7.18 (m, 2H), 6.90–6.80 (m, 4H), 6.26 (dd, J = 6.6, 6.6 Hz, 1H), 4.33–4.31 (m, 1H), 4.05–4.02 (m, 1H), 3.90–3.79 (m, 4H), 3.76 (s, 6H), 3.53 (s, 6H), 3.38–3.28 (m, 2H), 2.32–2.27 (m, 1H), 2.15–2.10 (m, 1H).

^{13}C NMR (126 MHz, CD_3OD, 300 K): δ 172.40, 162.97, 160.16, 151.10, 146.31, 137.16, 137.10, 131.36, 129.85, 129.38, 128.85, 127.86, 125.95, 114.18, 114.17, 87.72, 87.29, 86.38, 72.67, 65.31, 55.71, 54.41, 52.19, 40.51.

HRMS (ESI-TOF) *m/z*: $[M + Na]^+$ calcd for $C_{36}H_{39}N_3NaO_{11}$ 712.2477, found 712.2455.

Compound 6. To a solution of nucleoside **5** (146 mg, 0.212 mmol) in CH_2Cl_2 (1.3 mL) were added 2-cyanoethyl-*N,N,N′,N′*-tetraisopropylphosphorodiamidite (162 µL, 0.510 mmol, 2.4 eq), diisopropylamine (112 µL, 0.786 mmol, 3.7 eq), and tetrazole (55.1 mg, 0.786 mmol, 3.7 eq). After stirred at room temperature for 75 min, the reaction was quenched by the addition of benzyl alcohol polymer bound (321 mg, 0.321–0.642 mmol, 1.5–3.0 eq). After filtration to remove the polymer, the reaction mixture was concentrated under reduced pressure. Silica gel column chromatography ($CHCl_3$:CH_3OH = 100:0–10:1) gave the target compound **6** as a colorless foam (122 mg, 0.138 mmol, 65%, diastereomeric mixture). After characterized by ^{1}H and ^{31}P NMR spectroscopy and mass spectrometry, the phosphoramidite was immediately used for DNA synthesis.

^{1}H NMR (500 MHz, $CDCl_3$, 300 K): δ 7.98 (br, 1H), 7.47–7.43 (m, 2H), 7.34–7.18 (m, 8H), 6.85–6.80 (m, 4H), 6.27–6.23 (m, 1H), 4.54–4.48 (m, 1H), 4.16–4.12 (m, 1H), 3.88–3.69 (m, 11H), 3.66–3.58 (m, 3H), 3.55 (s, 6H), 3.40–3.32 (m, 2H), 2.61 (t, 1H, J = 6.4 Hz), 2.51–2.47 (m, 0.5H), 2.44–2.39 (m, 1.5H), 1.18–1.15 (m, 9.3H), 1.07 (d, 2.7H, J = 6.8 Hz).

^{31}P NMR (202 MHz, $CDCl_3$, 300 K): 149.23, 148.80 (diastereomers).

HRMS (ESI-TOF) *m/z*: $[M + Na]^+$ calcd for $C_{45}H_{56}N_5NaO_{12}P$ 912.3555, found 912.3588.

Determination of the molar extinction coefficient of the dcaU nucleoside. Fully deprotected nucleoside **4** was dissolved in 10 mM HEPES–NaOH buffer (pH 7.0) to prepare a 100-µM sample solution. UV–vis

absorption spectrum was measured on a V-730 spectrophotometer (JASCO) at 25 °C with a path length of 1 cm. According to the recorded absorbance at 260 nm, the molar extinction coefficient of **dcaU** nucleoside was determined to be 5.12×10^3 $M^{-1}\cdot cm^{-1}$.

Metal complexation study of the dcaU nucleoside. The sample solutions for UV–vis absorption measurement were prepared by mixing nucleoside **4** with varying concentrations of Gd^{III} ions in 10 mM HEPES–NaOH buffer (pH 7.0). The solution was incubated at 25 °C for at least 1 h prior to the measurement. UV absorption spectra were recorded on a V-730 spectrophotometer (JASCO) at 25 °C with a path length of 0.3 cm. The sample solution for ESI-TOF mass spectrometry was prepared by mixing nucleoside **4** with 0.5 equiv. of Gd^{III} ions in 10 mM NH_4OAc buffer (pH 7.0).

DNA synthesis. Oligonucleotides containing *N*,*N*-dicarboxymethyl-5-aminouracil (**dcaU**) nucleobases were synthesized on an Applied Biosystems 394 DNA synthesizer on a 1-μmol scale in a DMTr-on mode with ultramild deprotection phosphoramidites and reagents (Glen Research). DNA synthesis was performed according to the standard procedure except for an extended coupling time for **dcaU** (15 min). The products were cleaved from the solid support using a 0.3 M NaOH aqueous solution (1.0 mL) at room temperature for 2 h. After the further addition of a 0.3 M NaOH aqueous solution (1.0 mL), the supernatant was incubated at 37 °C for 22 h to remove protecting groups. The oligonucleotides were firstly detritylated and purified using a Glen-Pak cartridge (Glen Research) and further purified by reverse-phase HPLC (Waters XBridge Oligonucleotide OST C18 column, buffers: A = MeCN, B = 0.1 M TEAA (pH 7.0) + 2% MeCN, 60 °C). All DNA strands were identified by ESI-TOF mass spectrometry. Oligonucleotides not containing **dcaU** were purchased from Japan Bio Services after purified by HPLC and used without further purification. The amount of the oligonucleotides was determined based on the UV absorbance at 260 nm. The molar extinction coefficients (ε_{260}) of the oligonucleotides were calculated by the nearest-neighbor method ($\varepsilon_{260} = 5.12 \times 10^3$ $M^{-1}\cdot cm^{-1}$ for the **dcaU** nucleoside).

Strand **1**. 5′-CAC ATT A**dcaU**T GTT GTA-3′. HPLC retention time: 32.1 min (gradient: 4% A (0 min), 6.5% A (40 min)). ESI MS: *m*/*z* calcd for $[C_{151}H_{191}N_{51}O_{95}P_{14} - 4H]^{4-}$: 1167.51, found: 1167.45.

Strand **2**. 5′-TAC AAC A**dcaU**T AAT GTG-3′. HPLC retention time: 31.6 min (gradient: 4% A (0 min), 6.5% A (40 min)). ESI MS: *m*/*z* calcd for $[C_{151}H_{189}N_{57}O_{91}P_{14} - 4H]^{4-}$: 1171.70, found: 1171.96.

Duplex melting analysis. All samples were prepared by combining the oligonucleotides (2.0 μM each) in a 10 mM HEPES buffer (pH 7.0) containing 100 mM NaCl. After the addition of metal ions, the mixtures were annealed (from 60 °C to 4 °C, –1.0 °C/min) prior to the measurements. Absorbance at 260 nm (A_{260}) was recorded on UV-1800 and UV-1900 spectrophotometers (Shimadzu) equipped with a TMSPC-8 temperature controller. The temperature was raised from 4 °C to 60 °C at the rate of 0.2 °C/min. The melting curves were depicted after normalization as follows:

Normalized $A_{260} = \{A_{260}\ (t\ °C) - A_{260}\ (4\ °C)\} / \{A_{260}\ (60\ °C) - A_{260}\ (4\ °C)\}$.

The melting temperature (T_m) was determined as an inflection point of a melting curve using a T_m analysis

software LabSolutions (Shimadzu) with a 17-point adaptive smoothing program. Average T_m values of three independent runs are shown.

CD spectroscopy. The samples were prepared as described above. CD spectra were recorded on a JASCO J-820 spectropolarimeter with 10-time accumulation using a path length of 0.3 cm at 5 °C. The spectra were smoothed using a simple moving average smoothing program.

Metal-dependent regulation of the hybridization of dcaU-containing DNA strands. Native polyacrylamide gel electrophoresis (PAGE) was performed with FAM-labeled strand **2A′**. The samples were prepared by mixing strand **1**, **2**, and **2A′** (0.5 μM each) in 10 mM HEPES buffer (pH 7.0) containing 100 mM NaCl. The solutions were annealed (from 85 °C to 4 °C, –1.0 °C/min) in the absence or presence of Gd^{III} ions (1 equiv.). PAGE analysis was performed with 20% polyacrylamide gel in a cool incubator (4 °C). The bands were detected by FAM fluorescence and the yield of each product was calculated by comparing the intensities of the two bands observed in the same lane.

Time-course fluorescence analysis was performed with strand **2A′** containing a fluorescent 2-aminopurine (AP). The samples were prepared by mixing the DNA strands (0.5 μM each) in 10 mM HEPES buffer (pH 7.0) containing 100 mM NaCl. After annealed (from 85 °C to 20 °C, –1.0 °C/min), the samples were subjected to time-course fluorescence measurement at a constant temperature of 20 °C. The fluorescence intensity (λ_{ex} = 303 nm, λ_{em} = 371 nm) of AP was recorded in 30-second intervals. Gd^{III} (1 equiv.) and EDTA (5 equiv.) were alternately added at the defined time points during the measurement. The fluorescence intensity was normalized based on the emission intensity values of duplex **1**·**2A′** (I_{dsDNA}) and single strand **2A′** (I_{ssDNA}) as follows:

Normalized intensity = $(I_{obs} - I_{dsDNA}) / (I_{ssDNA} - I_{dsDNA})$.

2. Supplementary Figures

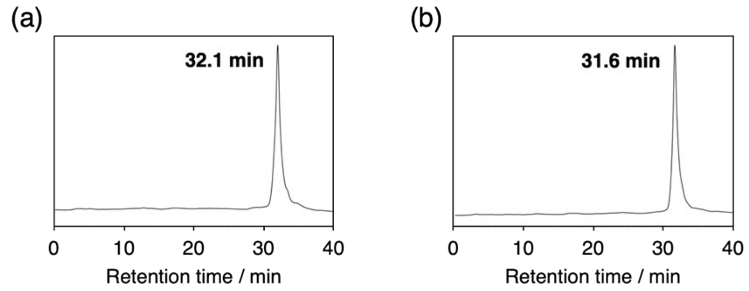

Fig. S1 HPLC analysis of DNA strands containing **dcaU** nucleotides after purification. (a) Strand **1** and (b) strand **2**. Waters XBridge Oligonucleotide OST C18 column (4.6 × 50 mm), flow rate: 1.18 mL/min, temperature: 60 °C, monitored at 260 nm. Gradient: 0 to 40 min, 4% A to 6.5% A (solvent A = MeCN, solvent B = 0.1M TEAA buffer (pH 7.0) + 2% MeCN).

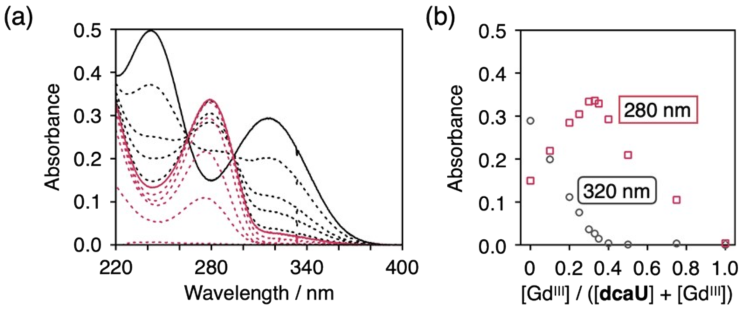

Fig. S2 Job's plot analysis of the metal complexation of **dcaU** nucleoside. (a) UV–vis absorption spectra of nucleoside **4** with the varying ratios of nucleoside **4** and Gd^{III} ions. [nucleoside **4**] + [Gd^{III}] = 200 μM, [Gd^{III}]/([nucleoside **4**] + [Gd^{III}]) = 0 (black solid line), 0.10, 0.20, 0.25, 0.30 (black dotted lines), 0.33 (red solid line), 0.35, 0.40, 0.50, 0.75, and 1.0 (red dotted lines) in 10 mM HEPES buffer (pH 7.0), *l* = 0.3 cm, 25 °C. (d) Plots of the absorbance at 280 nm and 320 nm against the ratios of [Gd^{III}]/([**dcaU**] + [Gd^{III}]).

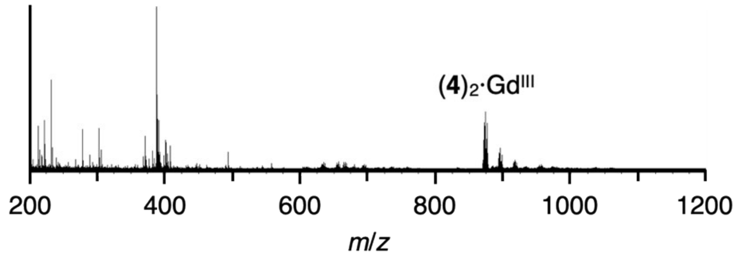

Fig. S3 ESI-TOF mass spectrum of nucleoside **4** with 0.5 equiv. of Gd^{III} ions. $(\mathbf{4})_2\cdot Gd^{III}$ = $C_{26}H_{34}N_6O_{18}P_{28}Gd$ (found: 874.07 (z = 1); calcd for $[(\mathbf{4})_2 + Gd^{III} + 2H]^+$: 874.10). [nucleoside **4**] = 100 μM, [Gd^{III}] = 50 μM in 20 mM NH_4OAc (pH 7.0). Positive mode. The magnified spectrum is shown in Fig. 2d.

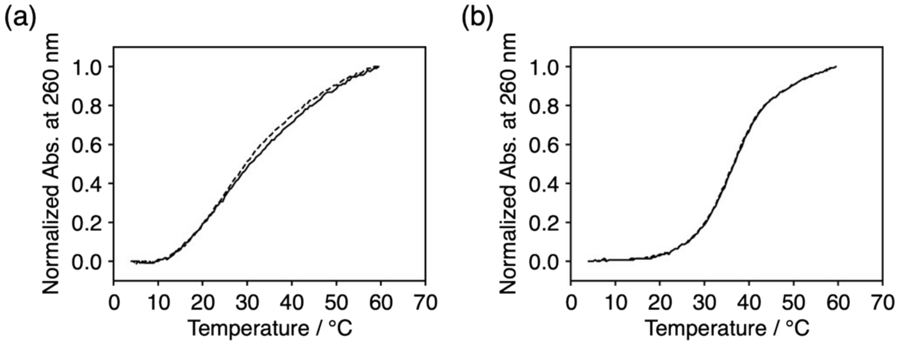

Fig. S4 (a) Melting curves of duplex **1**·**2T** containing a **dcaU**–T mismatch in the absence and presence of Gd^{III} ions. (b) Melting curves of duplex **1T**·**2T** containing a T–T mispair in the absence and presence of Gd^{III} ions. [duplex] = 2.0 μM in 10 mM HEPES buffer (pH 7.0) containing 100 mM NaCl, 0.2 °C/min. [Gd^{III}]/[duplex] = 0 (solid line) and 1 (dotted line).

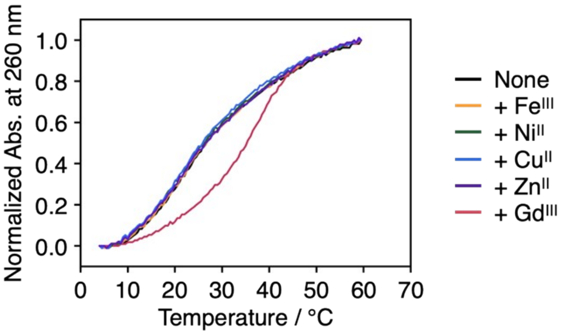

Fig. S5 Melting curves of duplex **1**·**2** containing a **dcaU**–**dcaU** mismatch in the presence of various metal ions. [duplex] = 2.0 μM in 10 mM HEPES buffer (pH 7.0) containing 100 mM NaCl, 0.2 °C/min. [metal ion]/[duplex] = 1.

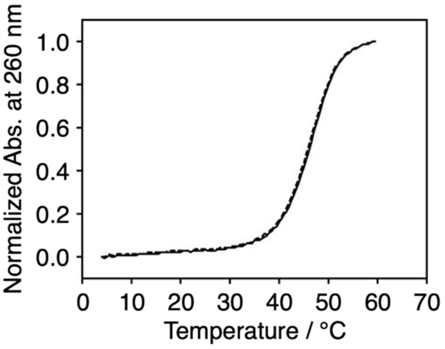

Fig. S6 Melting curves of duplex **1T**·**2A** containing a T–A pair in the absence and presence of Gd^{III} ions. [duplex] = 2.0 μM in 10 mM HEPES buffer (pH 7.0) containing 100 mM NaCl, 0.2 °C/min. [Gd^{III}]/[duplex] = 0 (solid line) and 1 (dotted line).

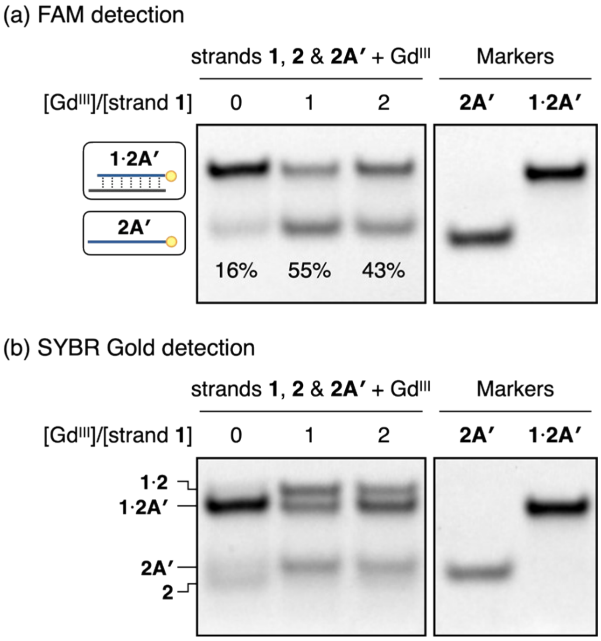

Fig. S7 Native PAGE analysis of an equimolar mixture of strands **1**, **2**, and **2A′** in the absence and presence of Gd^{III} ions (1 equiv.). (a) FAM detection, (b) SYBR Gold detection. [DNA] = 0.5 µM each in 10 mM HEPES buffer (pH 7.0) containing 100 mM NaCl. The samples were annealed prior to the analysis. Strand **2A′** was labeled with FAM for detection. 20% gel at 4 °C. The yields were calculated from the average of three independent experiments.

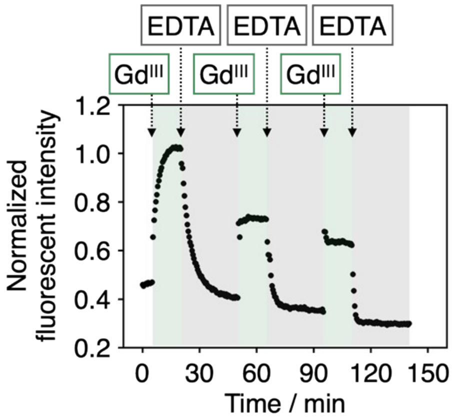

Fig. S8 Reversible regulation of the hybridization of **dcaU**-containing strand **1** by the sequential addition of Gd^{III} ions and EDTA. After annealing the mixture of the three strands (**1**, **2** and **2A′**), Gd^{III} (1 equiv.), EDTA (5 equiv.), Gd^{III} (5 equiv.), EDTA (25 equiv.), Gd^{III} (25 equiv.), and EDTA (125 equiv.) were sequentially added. λ_{ex} = 303 nm, λ_{em} = 371 nm, 20 °C. [DNA] = 0.5 µM each in 10 mM HEPES buffer (pH 7.0), 100 mM NaCl.

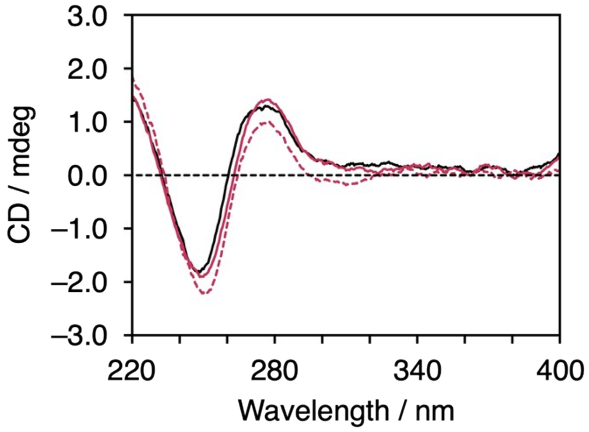

Fig. S9 CD spectra of duplex **1**·**2** containing a **dcaU**–**dcaU** mismatch. [duplex] = 2.0 μM, [Gd^{III}]/[duplex] = 0 (black solid line), 1 (red solid line), and 2 (red dotted line) in 10 mM HEPES buffer (pH 7.0), 100 mM NaCl, l = 0.3 cm, 5 °C.

3. NMR Spectra

Compound 3

^1H NMR (500 MHz, 300 K, CD_3OD).

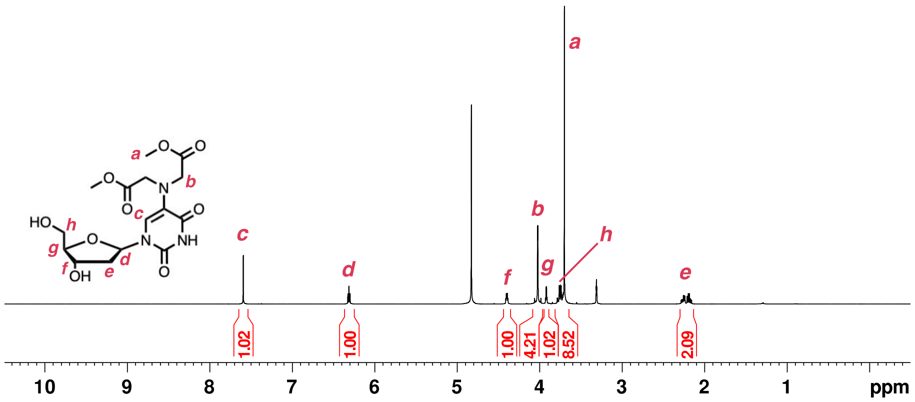

^{13}C NMR (126 MHz, 300 K, CD_3OD).

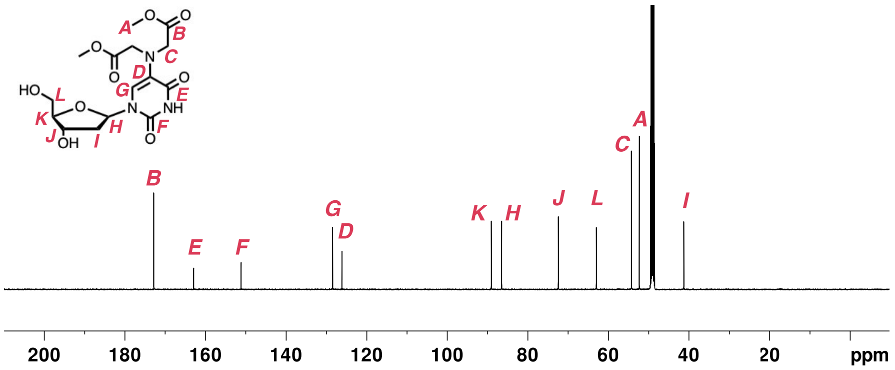

Compound 4·Na^+_2

^1H NMR (500 MHz, 300 K, CD_3OD).

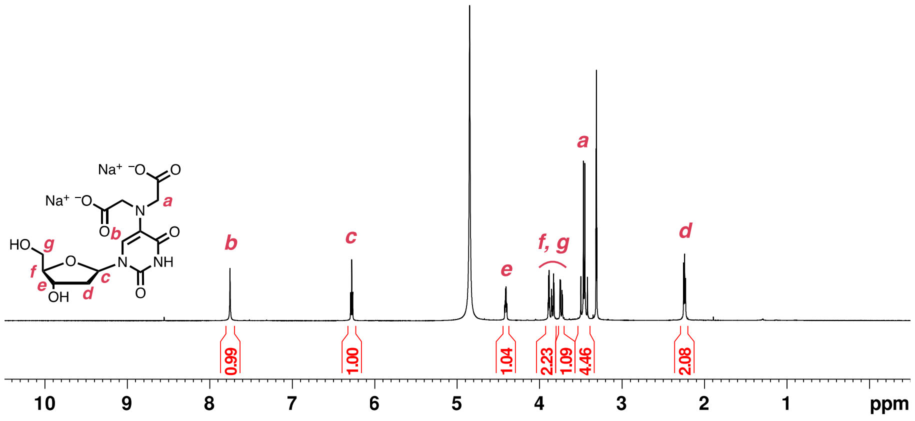

^{13}C NMR (126 MHz, 300 K, CD_3OD).

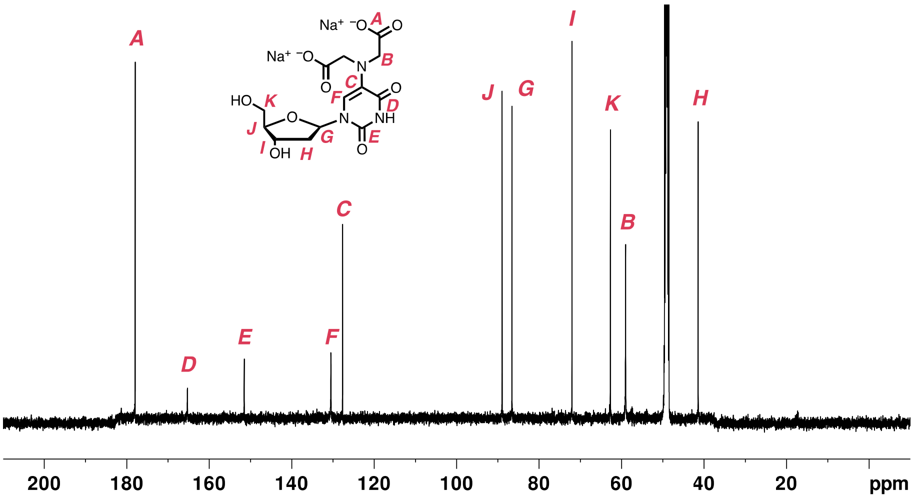

Compound 5

^{1}H NMR (500 MHz, 300 K, CD_3OD).

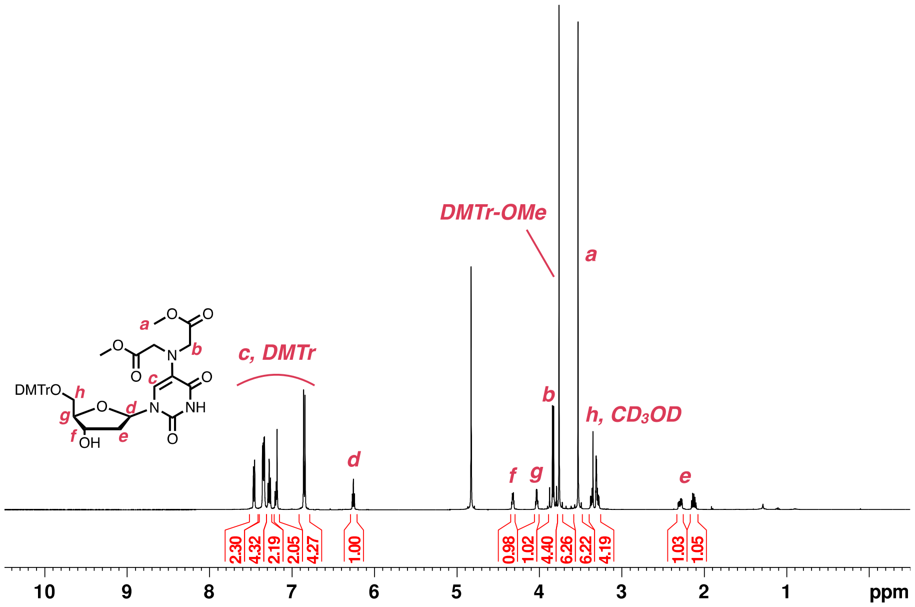

^{13}C NMR (126 MHz, 300 K, CD_3OD).

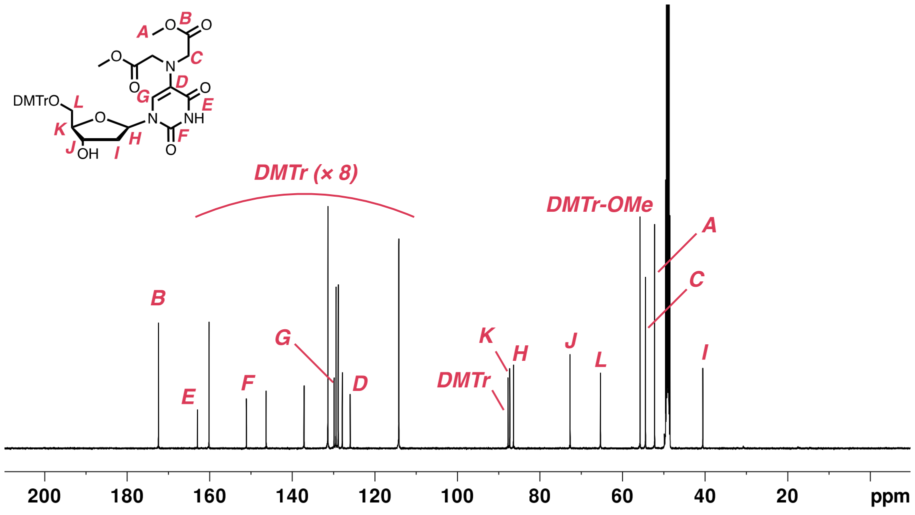

Compound 6

^{1}H NMR (500 MHz, 300 K, $CDCl_3$).

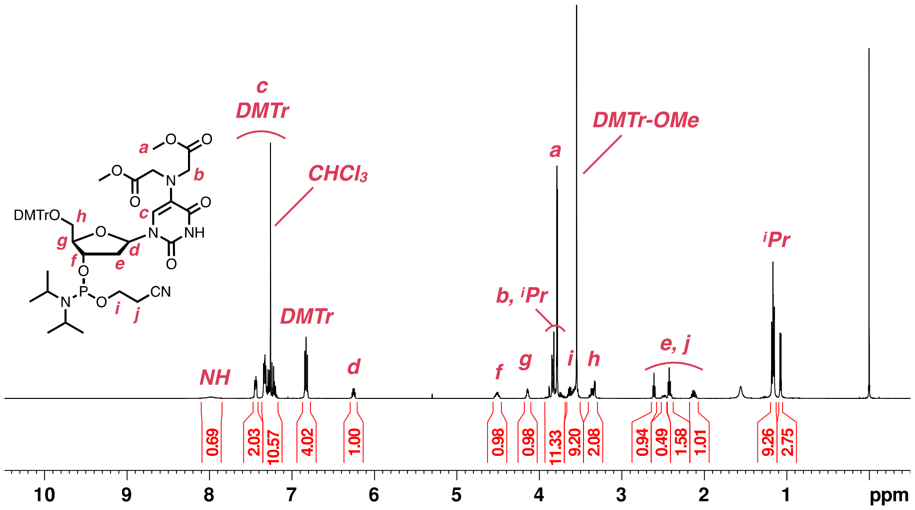

^{31}P NMR (202 MHz, 300 K, $CDCl_3$).

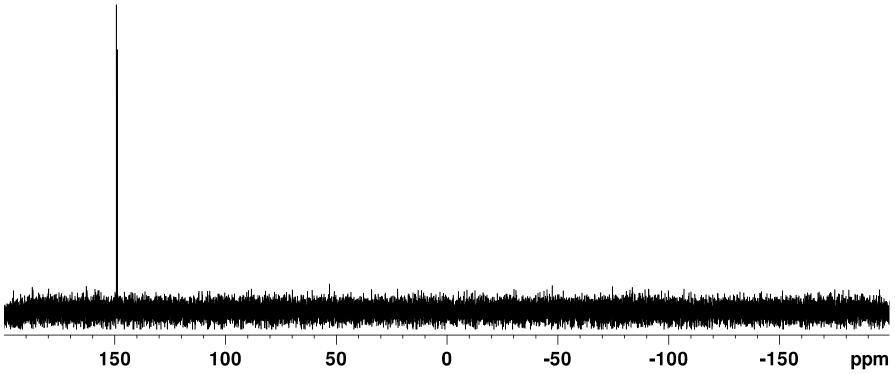

Supplement: SC-014-D2SC06534G-s001 [file SC-014-D2SC06534G-s001.pdf]
